# Predicting the mortality of smoking attributable to cancer in Qingdao, China: A time-series analysis

**DOI:** 10.1371/journal.pone.0245769

**Published:** 2021-01-25

**Authors:** Fei Qi, Zhenshi Xu, Hua Zhang, Rui Wang, Yani Wang, Xiaorong Jia, Peng Lin, Meiyun Geng, Yiqing Huang, Shanpeng Li, Jun Yang

**Affiliations:** 1 Qingdao Municipal Center for Disease Control and Prevention, Qingdao, Shandong, China; 2 Qingdao Mental Health Center, Qingdao, Shandong, China; 3 Medical College, Qingdao University, Qingdao, Shandong, China; 4 Qingdao Municipal Health Commission, Qingdao, Shandong, China; The Ohio State University, UNITED STATES

## Abstract

Smoking is the leading preventable cause of death and disability from cancer in China. To provide a scientific basis for tobacco control strategies and measures, this study investigated cancer deaths attributed to smoking from 2005 to 2017 and predicted mortality trends from 2018 to 2020 in Qingdao. We used time series analysis to evaluate the number of deaths attributed to smoking among residents over 35 years old in Qingdao and predicted mortality trends. The number of cancer deaths attributed to smoking in Qingdao from 2005 to 2016 was between 170 and 407, showing an upward trend and a certain periodicity. The best model is the ARIMA (2,1,0)×(3,1,0)_12_, with the lowest BIC (6.640) and the highest stationary R^2^ (0.500). The predicted cancer deaths curve attributed to smoking in 2017 is consistent with the actual curve, with an average relative error of 5.74%. Applying this model to further predict the number of cancer deaths attributed to smoking in Qingdao from January 2018 to December 2020, the predicted results were 5,249, 5,423 and 6,048, respectively. The findings emphasized the need to further strengthen tobacco control measures to reduce the burden of disease caused by tobacco.

## Introduction

China is the largest producer and consumer of tobacco in the world, with a current smoking rate of 27.7% (52.1% for men, 2.7% for women), and the number of current smokers reaching 316 million [1]. In 2014, China's tobacco consumption accounted for 44% of the world's total, surpassing the total of 29 countries with higher tobacco consumption such as Indonesia, Japan, Russia and the United States [2]. At present, 1.4 million people die of tobacco-related diseases every year in China [3]. This number is expected to exceed 3 million by 2050, which has brought huge economic and health losses to China [3, 4]. The number of years of potential life lost (YPLL) caused by smoking in China was 7.67 million years in 2014, which means that each death caused by smoking was reduced by 15 years. Furthermore, smoking has gained attention not only because of its harmful effect on health, but also because it may place a huge burden on the economy, which may increase significantly over the coming decades. In the same year, the economic losses caused by smoking in China totaled about 350 billion CNY, accounting for 0.55% of the annual GDP [2].

Smoking is one of the main reasons for the rapid increase in the number of deaths from noncommunicable diseases in China. Diseases such as cardiovascular disease, cancer, respiratory diseases and diabetes have become the primary threat to the health of our residents. In 2012, the number of people that died from non-communicable diseases was about 8.6 million [5]. Among them, cancer was the most serious, and its mortality has surpassed cardiovascular disease since 2010 and has become the leading cause of death in the Chinese population [6]. Findings from a national study have shown that 32.7% and 5% of male and female cancer deaths in China are caused by smoking [7]. In addition, more than 160000 people die from cancer each year, and 20%-30% of deaths are caused by smoking [8]. And if the number of smokers continues to decrease, lung cancer mortality is expected to drop by 79% in the next 30 years [9]. This clearly shows that the tobacco threat poses a huge public health challenge.

An earlier study has shown that the disease burden caused by smoking in China has significantly increased in the past few decades [10]. In order to reduce smoking rates, cities in China have formulated relevant tobacco control measures. However, the quality of the implementation and enforcement of smoke-free laws varies among cities. Qingdao has enacted its smoke-free law in 2013, which prohibited smoking in certain public places. Nevertheless, the current status of tobacco smoking in Qingdao remains serious. According to the 2014 survey results on tobacco smoking in Qingdao, the current smoking rate of residents over the age of 15 is 21.36%, with 40.5% for men. In 2015, there were 14,238 cancer deaths in Qingdao, of which 4,148 were due to smoking, accounting for 29.1% of the total cancer deaths [11]. The life expectancy loss of residents in Qingdao caused by cancer deaths due to smoking was 0.87 years, with 1.19 years for men and 0.45 years for women [12]. Hence, the cancer burden due to smoking in Qingdao cannot be ignored.

In this study, we aim to establish a time series model to evaluate the number of cancer deaths attributed to smoking in Qingdao from 2005 to 2016, and to project the trend of cancer deaths caused by smoking from 2017 to 2020 in Qingdao. We hypothesized that the current number of cancer deaths due to smoking will show an upward trend and continue to rise in the coming years. In addition, we would like to show that this research can fill the gaps in the literature on the trend of cancer deaths caused by smoking in Qingdao, and provide a basis for further development of tobacco control strategies and measures.

## Materials and methods

### Data sources

Based on the 2013 Global Burden of Disease Study (GBD), this study included 12 tobacco-related cancers, including esophageal cancer, gastric cancer, liver cancer, lung cancer, colorectal cancer, oral cancer, nasopharyngeal cancer, pancreatic cancer, kidney cancer, bladder cancer, leukemia, and cervical cancer. The relative risk (RR) of these 12 cancers also comes from the relevant studies of GBD 2013 [13]. The RRs for the 12 major smoking-related cancers and ICD-10 (International Classification of Diseases, 10th Revision) codes are shown in Table 1.

**Table 1:** Relative risks and diagnostic codes for 12 major smoking-related cancers.

ICD-10 Codes	Diseases	Men	Women
**C15**	Esophageal cancer	6.676	6.357
**C16**	Stomach cancer	1.927	1.57
**C22**	Liver cancer	2.540	1.724
**C33,C34**	Lung cancer	22.511	14.095
**C18-C21**	Colorectal cancer	1.325	1.418
**C00-C08**	Oral Cancer	8.162	6.056
**C11**	Nasopharynx cancer	8.227	6.089
**C25**	Pancreatic cancer	2.506	2.098
**C64**	Kidney cancer	2.293	1.518
**C67**	Bladder Cancer	3.332	2.582
**C91-C95**	Leukemia	2.013	1.163
**C53**	Cervical cancer		1.679

The cancer death data from 2005 to 2017 was derived from the death cause reporting system of the Qingdao Municipal Center for Disease Control and Prevention. The system covers the household registration population of 10 districts and cities under the jurisdiction of the Qingdao Municipality. The population composition information and data for 2005–2017 comes from the Qingdao Municipal Public Security Bureau. The scope of this analysis was limited to adults older than 30 years, as most cumulative hazardous effects of smoking-related cancers are unlikely to manifest in individuals younger than middle age. Stratification of the population by sex and age is shown in S1 Table. Based on previous research methods [14, 15], we estimated the number of cancer deaths attributed to smoking in Qingdao from 2005 to 2017. Since the data on smoking rate in the past few years is not comprehensive, and the current smoking prevalence is not enough to evaluate the cumulative harm of smoking, we can use the smoking impact ratio (SIR) to estimate cancer mortality indirectly. SIR can reflect the cumulative harm of smoking, therefore, indirect estimation of smoking attributable mortality can reflect the impact of current and past smoking on disease mortality for cancer, COPD and other diseases. In the absence of direct information about smoking history, this method can directly estimate the cancer mortality caused by smoking from the obtained statistical data of disease mortality. The proportion of deaths caused by smoking is estimated using the standard population attributable fraction (PAF). SIR was used to measure cumulative hazards of smoking among the population in Qingdao and to calculate PAFs. The formula for PAF and the smoking-attributable mortality are as follows:

$$PAF=\frac{SIR(RR-1)}{SIR(RR-1)+1}$$

SAM=PAF×M

where RR represents relative risk and M represents deaths related to cancers caused by smoking.

### Method

The Autoregressive Integrated Moving Average ARIMA model is a well-known time series prediction method proposed by Box and Jenkins in the early 1970s, and also called the Box-Jenkins model method [16]. It comprehensively considers the trend change, periodic variation and random interference of the sequence, and fully extracts the available information in the data. It is one of the more accurate and universal time series analysis methods. The ARIMA model is a combination of multiple models, including the Autoregressive model, the Moving average model, and the Autoregressive Moving Average model. The form of the ARIMA model is represented by ARIMA (p, d, q), where p is the autoregressive order, d is the number of differences, and q is the moving average order.

When the sequence has both short-term correlation and periodicity, the d-order trend difference is difficult to smooth the sequence, and the original sequence needs to be seasonally differentiated with period s to eliminate its periodicity. The seasonal difference is the difference between the observed value at a certain time in the sequence and the observed value at the same time as the previous period. Since there is a product relationship between the short-term correlation and the seasonal effect, the sequence that satisfies this condition can be represented by the model ARIMA (p, d, q) × (P, D, Q) s, where p is the non-seasonal autoregressive order, d is a non-seasonal difference order, q is a non-seasonal moving average order, P is a seasonal autoregressive order, D is a seasonal difference order, Q is a seasonal moving average order, and s is a seasonal period length.

Four steps are required to make predictions using the ARIMA model. First, the application of the ARIMA model requires a sequence of stationary non-white noise sequences. If the sequence is a non-stationary sequence, natural logarithmic transformation, differential and seasonal difference processing are performed on the original data, and d and D are determined according to the number of differences. Second, p, q, P, and Q are initially determined based on the autocorrelation function (ACF) image and the partial autocorrelation function (PACF) image of the processed stationary time series. Third, the least squares estimation or maximum likelihood method is used to estimate the value of unknown parameters in the model. The significance of each parameter is tested, and the residual sequence is subjected to a white noise test. If the model is not suitable, we return to the model identification stage to re-select the model; if there are multiple effective models, we select according to the fitting goodness index which includes the coefficient of determination (R^2^) and normalized Bayesian Information Criterion (BIC). Finally, the determined model is applied for analysis and prediction.

## Results

### Model identification

**Fig 1:**
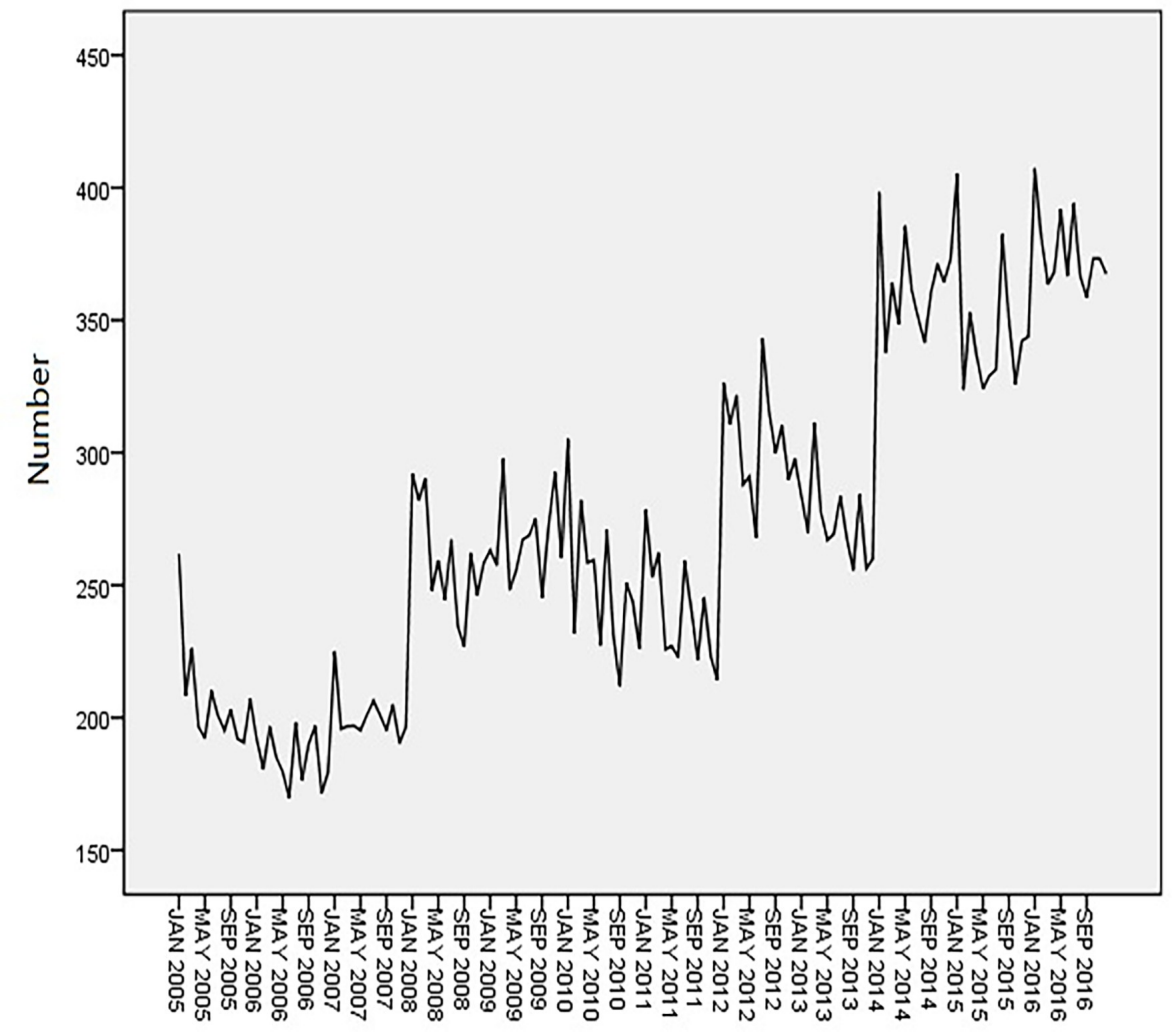
Monthly smoking-related cancer deaths from January 2005 to December 2016 in Qingdao.

**Fig 2:**
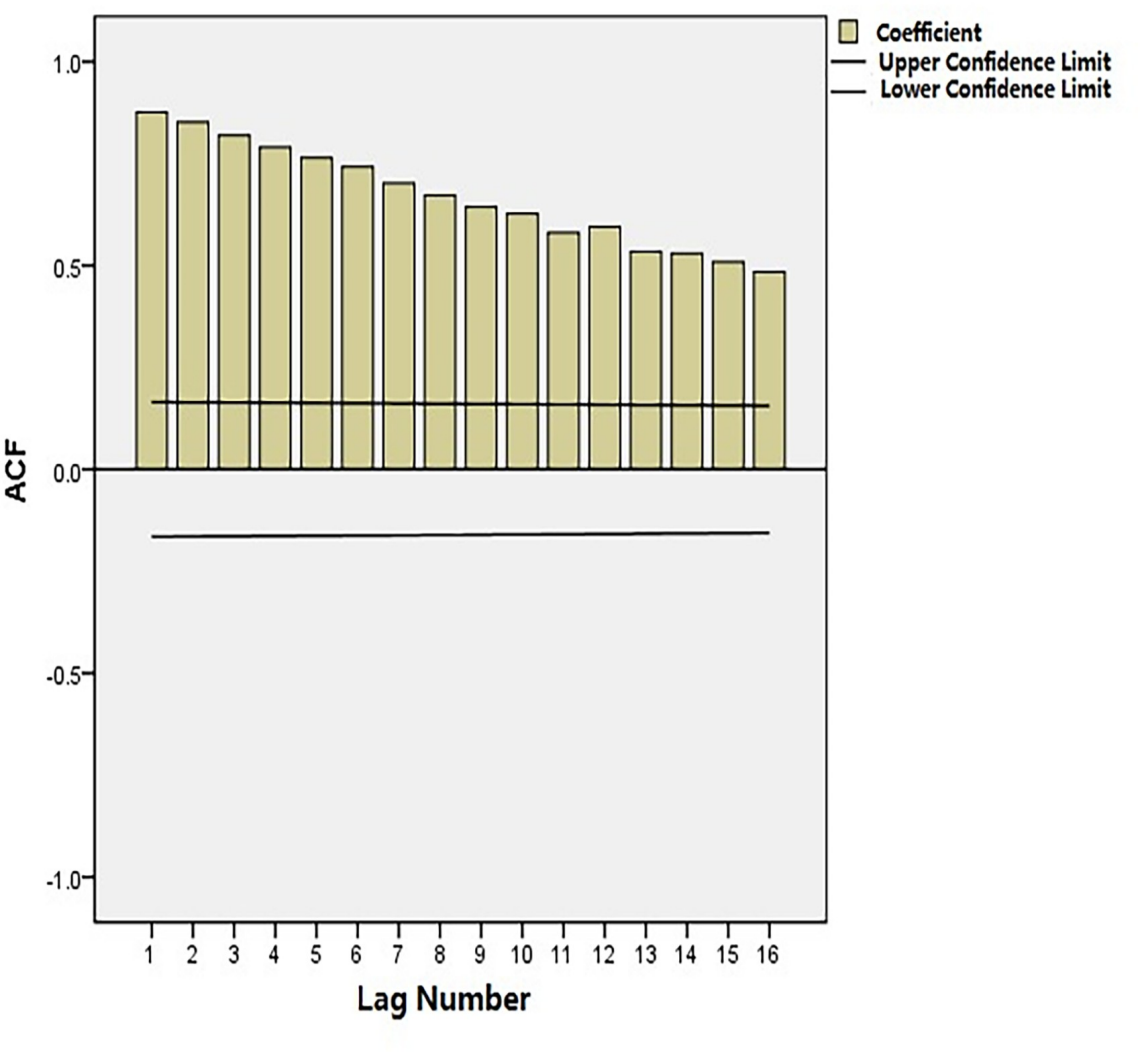
Autocorrelation function (ACF) of the monthly smoking-related cancer deaths.

**Fig 3:**
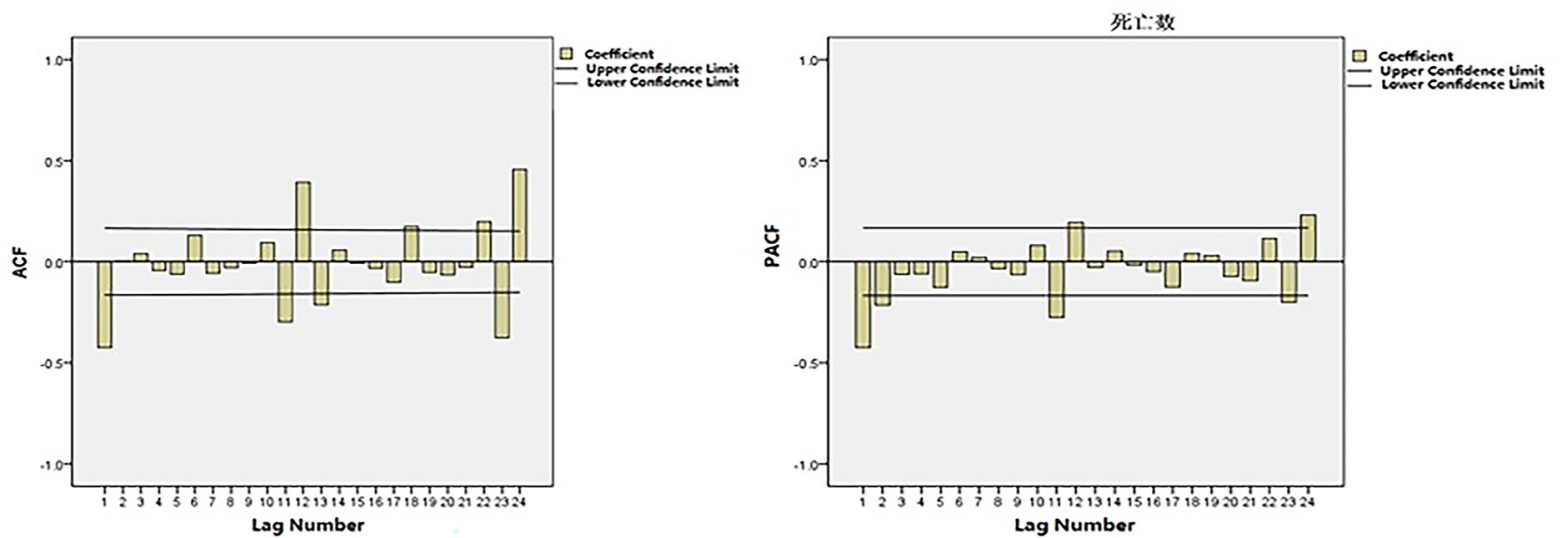
ACF and PACF of the natural logarithmic transformation of deaths after 1-step non-seasonal differences.

**Fig 4:**
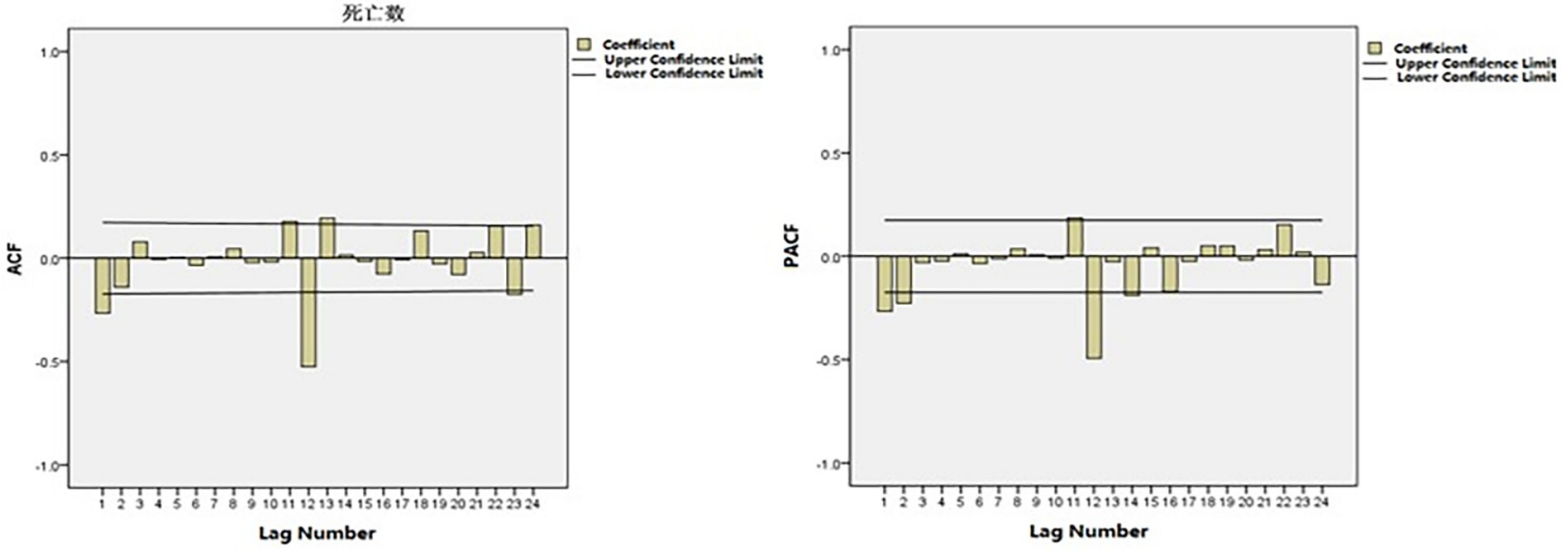
ACF and PACF of the natural logarithmic transformation of deaths after 1-step non-seasonal and 1-step seasonal differences.

Time-series data from January 2005 to December 2016 was used as the training set. The total number of cancer deaths attributed to smoking in Qingdao from 2005 to 2017 was 43628, and the number of deaths in each year was 2484, 2215, 2406, 3111, 3204, 2999, 2874, 3662, 3286, 4385, 4184, 4512 and 4369. The annual average number of deaths is 3356, and the average annual growth rate is 4.82%. Fig 1 shows the monthly smoking-related cancer deaths from January 2005 to December 2016 in Qingdao, which reveals an upward trend. Fig 2 shows that the ACF of the original data had a non-stationary variance. Natural logarithmic transformation was performed on the data and the first-order difference was taken to eliminate the effects of the linear trend. Fig 3 shows that that the ACF and PACF of the differentiated data still had the effects of seasonal trends and needed seasonal difference. Fig 4 shows that the ACF and PACF of the new data tended to be stationary after using the first-order seasonal difference, which determined that d and D in the ARIMA (p, d, q) × (P, D, Q)_12_ have values of 1 and 1, respectively.

### Model diagnosis

First, the characteristics of the autocorrelation coefficient and partial autocorrelation coefficient (Fig 4) within the 12th order of the lag period was observed. Based on auto-correlation coefficient tailing and partial auto-correlation coefficient second-order truncation, it is judged that p = 2, q = 0. Second, the characteristics of the autocorrelation coefficient and partial autocorrelation coefficient at the periodic node were then observed to determine the seasonal parameters P and Q. It is judged that the autocorrelation coefficient is tailed and partial autocorrelation coefficient is censored, that is, Q = 0, and the model is further estimated as ARIMA (2,1,0) × (P,1,0)_12_. In most cases, the order of P does not exceed 3, so the method P is used to determine the parameter P one by one from the low order to the high order. Therefore, the alternative models are: ARIMA (2, 1, 0) × (1, 1, 0)_12_, ARIMA (2, 1, 0) × (2, 1, 0)_12_, ARIMA (2, 1, 0) × (3,1,0)_12_. Table 2 shows the parameter estimation for plausible ARIMA models.

**Table 2:** Parameter estimation for plausible ARIMA models.

	ARIMA (2,1,0)×(1,1,0) _12_			ARIMA (2,1,0)×(2,1,0) _12_			ARIMA (2,1,0)×(3,1,0) _12_		
	value	t	p	value	t	p	value	t	p
**AR (1)**	-0.256	-3.022	0.003	-0.308	-3.511	0.001	-0.344	-4.034	0.000
**AR (2)**	-0.310	-3.639	0.000	-0.289	-3.333	0.001	-0.328	-3.822	0.000
**SAR (12)**	-0.591	-8.163	0.000	-0.755	-8.434	0.000	-0.847	-9.753	0.000
**SAR (24)**	-	-	-	-0.281	-2.982	0.003	-0.586	-5.558	0.000
**SAR (36)**	-	-	-	-	-	-	-0.449	-5.013	0.000
**Ljung-Box Q**	-	-	0.977	-	-	0.976	-	-	0.969
**Stationary R**^**2**^	0.394			0.427			0.500		
**R**^**2**^	0.804			0.818			0.843		
**BIC**	6.770			6.740			6.640		

Based on the results of the goodness-of-fit test statistics, we confirmed the optimal ARIMA (2,1,0)×(3,1,0)_12_ model, which had the highest stationary R^2^ (0.500), lowest BIC (6.640) among the three plausible models.

### Forecast and analysis

**Fig 5:**
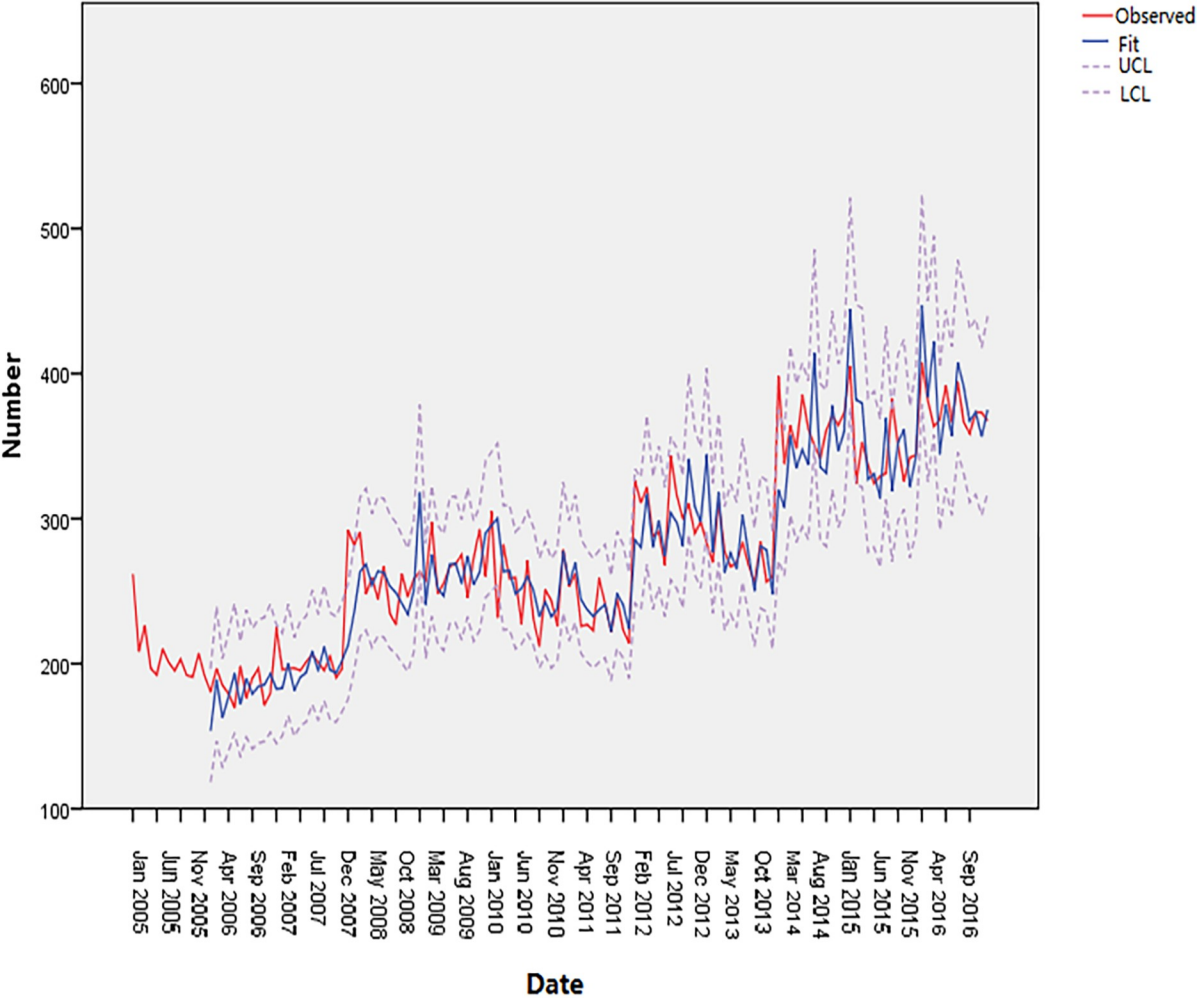
Observed value of the ARIMA (2,1,0) × (3,1,0)_12_ model.

The ARIMA (2,1,0) × (3,1,0)_12_ model was used to fit the number of cancer deaths attributable to smoking in Qingdao from January 2005 to December 2016. Fig 5 shows that the predicted curve is consistent with the actual curve, and the actual curve is within the 95% confidence interval.

The model was used to predict the number of cancer deaths attributed to smoking in Qingdao in 2017, and the actual value is used to test the fit of the model. Table 3 shows that the 95% confidence interval of the predicted value includes the actual value, with the relative error range from 0.25% to 16.18%, and the average relative error is 5.74%, which proves that the model fits well.

**Table 3:** Comparison of the predicted and actual values of the ARIMA (2,1,0) × (3,1,0)_12_ model.

Month	Forecast	Actual death	Absolute error	relative error (%)	95%CI	
					LCL	UCL
**1**	400	401	-1	0.25	339	469
**2**	363	332	31	9.34	297	438
**3**	395	340	55	16.18	319	483
**4**	369	358	11	3.07	291	462
**5**	369	397	-28	7.05	284	471
**6**	365	344	21	6.10	276	474
**7**	377	350	27	7.71	280	497
**8**	376	379	-3	0.79	275	503
**9**	362	353	9	2.55	260	492
**10**	377	354	23	6.50	267	518
**11**	364	360	4	1.11	254	507
**12**	367	400	-33	8.25	252	518

This model was used to further predict the number of cancer deaths due to smoking in Qingdao from January 2018 to December 2020; the results are shown in Table 4. The number of cancer deaths attributed to smoking in Qingdao shows an upward trend in the next three years; the predicted results are 5,249, 5,423 and 6,048 respectively. In addition, we can conclude that the predicted annual growth rate of deaths is 7.34%, and the number of deaths caused by smoking shows an upward trend compared with the past 11 years.

**Table 4:** The number of cancer deaths due to smoking from January 2018 to December 2020.

Month	2018	95%CI		2019	95%CI		2020	95%CI	
		LCL	UCL		LCL	UCL		LCL	UCL
**1**	477	321	685	501	277	839	541	240	1060
**2**	417	275	607	430	233	730	490	213	973
**3**	437	284	645	458	244	788	497	212	1000
**4**	424	270	634	440	230	768	489	205	998
**5**	449	282	680	441	226	780	512	210	1058
**6**	429	265	659	436	220	782	491	198	1027
**7**	435	265	675	448	221	812	515	203	1089
**8**	430	258	676	472	230	868	502	194	1075
**9**	431	255	685	449	215	836	492	187	1067
**10**	440	257	707	444	209	836	507	189	1111
**11**	438	252	711	451	208	858	506	186	1122
**12**	442	251	726	453	206	872	506	182	1134
**Total**	5249	-		5423	-		6048	-	

LCL, Lower confidence limit; UCL, upper confidence limit.

## Discussion

In an analysis of the data based on the number of cancer deaths attributed to smoking, we used ARIMA models to fit and predict the changing trends of smoking-attributable cancer deaths from 2005 to 2017. A prior study using data from 2005 to 2016 found that cancer deaths attributed to smoking showed a rising trend in Qingdao [14]. Our study added to the previous study by extending the analysis to 2020, and the results showed that the forecast results matched the actual data in 2017 well with an average relative error of 5.74%. According to the testing results, we found that the conducted model ARIMA (2,1,0) × (3,1,0)_12_ was reliable with high validity and can be used to forecast the expected number of cases. Further prediction for the number of cancer deaths attributed to smoking from 2018 to 2020 showed predicted results are 5,249, 5,423 and 6,048 respectively.

The model projected an increase in the number of cancer deaths attributed to smoking in Qingdao from 2018 to 2020. The model assumed that the current trend in smoking will persist for several years. Because of the higher smoking prevalence among residents aged 45–69 years [17], which is considered a high-risk group suffering from smoking-related disease [18], and the lag time between smoking and related cancers, the trend of deaths from cancer caused by smoking will continue to increase over time. This was consistent with the conclusion of another relevant study in China [10], which indicated that the smoking-related deaths will enter a high incidence period in 2010 to 2030 through five national smoking prevalence surveys in 1984, 1996, 2002, 2010 and 2015.

Reducing the smoking rate can effectively reduce the number of deaths attributed to smoking in the future. A previous study found that 326,000 deaths due to noncommunicable diseases (e.g., cancer, cardiovascular diseases, diabetes mellitus and chronic respiratory diseases), including 222,400 cancer-related deaths among 30–70-year-old Chinese residents by 2030 could be avoided if there is a 30% reduction in smoking rate from the 2013 level [19]. Differences in socioeconomic status [20], disparities in educational level [20], changes in the prospects of tobacco products (e-cigarette and compound tobacco use) [21, 22] will contribute to the rise in smoking prevalence. Several studies around the world indicated that effective tobacco control measures can help reduce smoking prevalence, which in turn would reduce the disease burden caused by smoking. Taiwan has adopted a series of tobacco control policies which show that it can reduce the burden of disease caused by smoking and second-hand smoke exposure effectively, and has experienced a decreased trend in disease burden from 1990 to 2013 [23]. A study in the United States which used models to predict the number of cancer deaths caused by smoking also showed a downward trend [9]. Furthermore, the American Cancer Society's 2018 Cancer Statistics Report indicated that tobacco use in the United States has decreased due to increased awareness of tobacco harm and the implementation of comprehensive tobacco control measures, and the mortality rates of lung cancer had fallen by 45% from 1990 to 2015 in men and 19% from 2002 to 2015 in women [24].

This information illustrates the importance of implementing effective tobacco control policies. The implementation of a comprehensive tobacco control policy in 27 European Union countries is the key to reducing smoking prevalence and increasing the smoking cession rates [25]. Qingdao has enacted the smoke-free law on 1 September 2013. This law prohibits smoking in restaurants, bars and hotels, and does not allow designated smoking rooms in these venues, which makes it more comprehensive than the laws implemented in other cities in China [26]. However, the enforcement of the smoke-free law is relatively poor; although it provides severe penalties for violations, no fines have been issued within the first year of law enforcement [27]. Moreover, the lag time between the implementation of tobacco control measures (smokers quitting or young people never starting to smoke) and the decrease in smoking-related cancer mortality is exceptionally long because smoking-related cancers develop after approximately 20–30 years of continuous smoking; therefore, the number of cancer deaths attributed to smoking in Qingdao remain on an upward trend in the next 3 years. The government of Qingdao should strengthen the implementation of this regulation to create a smoke-free environment. The Smoke-free law will help people reduce the tobacco use and protect them against the health risks of tobacco smoke. Surveys indicated that cigarette consumption in the United States is between 5% and 20% lower per capita in states with comprehensive smoke-free laws. Exposure to tobacco smoke is proven to cause heart disease, cancer and many other diseases. In many countries, it causes more than 10% of all tobacco-related deaths. The government of Qingdao should strengthen the implementation of this regulation to reduce the overall disease burden caused by smoking, as this would help not only to reduce the burden of cancer, but also other non-communicable diseases and some communicable diseases.

To the best of our knowledge, this is the first study to predict the number of cancer deaths attributed to smoking in Qingdao from 2017 to 2020 using the ARIMA model. These findings are valuable for the implementation and assessment of tobacco control measures and changes in the disease burden caused by smoking. However, the limitations of the study must be noted. First, we only analyzed the data of cancer deaths attributed to smoking from 2005 to 2016 in Qingdao. The results predicted were relatively accurate in the next 1–2 years. However, the 95% confidence interval was obviously wide in the third year, showing poor prediction. In order to ensure the accuracy of the prediction model, monitoring data needs should be added continually to the sequence over time. Second, our analysis only included the smoking-related cancer burden attributed to cigarette smoking, and it does not include cancer burden from former smokers and second-hand smokers, or the use of e-cigarettes and other tobacco types. Further research should consider the disease burden of former and passive smokers and various tobacco types. Finally, the findings may not be generalized to other cities in China. The enforcement of the smoke-free law in Qingdao is not as strict as in other cities, and different impacts may be observed in cities with strong tobacco control.

## Conclusion

The number of cancer deaths attributed to smoking in Qingdao from 2005 to 2017 showed a fluctuating upward trend. The ARIMA (2,1,0) × (3,1,0)_12_ model is relatively accurate in predicting the number of cancer deaths caused by smoking in Qingdao, and the prediction results from 2008 to 2020 are 5,249, 5,423 and 6,048, respectively. Strengthening tobacco control measures to reduce smoking rates and increase smoking cessation rates is of great significance for reducing the disease burden of malignant tumors in Qingdao.

## Supporting information

S1 Table The population of Qingdao by sex and age in form 2005 to 2017. (XLSX)

## References

[1] Liang X. China Adult Tobacco Survey Report 2015. Beijing: People's Medical Publishing House; 2016.

[2] World Health Organization. The bill China cannot afford: health, economic and social costs of China's tobacco epidemic Manila, Philippines: Publications Office, World Health Organization, Regional Office for the Western Pacific; 2017.

[3] Chen Z, Peto R, Zhou M, Iona A, Smith M, Yang L, et al Contrasting male and female trends in tobacco-attributed mortality in China: evidence from successive nationwide prospective cohort studies. Lancet (London, England). 2015;386(10002):1447–56. 10.1016/S0140-6736(15)00340-2 . 26466050 PMC4691901

[4] Yang G, Wang Y, Wu Y, Yang J, Wan X. The road to effective tobacco control in China. Lancet (London, England). 2015;385(9972):1019–28. 10.1016/S0140-6736(15)60174-X . 25784349

[5] Wang SY, Langenbrunner J.C., Marquez P.V. Toward a healthy and harmonious life in China: stemming the rising tide of non-communicable diseases. China: World Bank, 2011.

[6] National Bureau of Statistics of China. China Statistical Yearbook, 2010. Beijing: China Statistics Press; 2010.

[7] Jianbing W, Yong J, Wenqiang W, Gonghuan Y, Youlin Q, Boffetta, et al Estimation of cancer incidence and mortality attributable to smoking in China. Cancer causes & control: CCC. 2010;21(6):959–65. 10.1007/s10552-010-9523-8 . 20217210

[8] Lortet-Tieulent J, Goding Sauer A, Siegel RL, Miller KD, Islami F, Fedewa SA, et al State-Level Cancer Mortality Attributable to Cigarette Smoking in the United States. JAMA Intern Med. 2016;176(12):1792–8. 10.1001/jamainternmed.2016.6530 . 27775761

[9] Jeon J, Holford TR, Levy DT, Feuer EJ, Cao P, Tam J, et al Smoking and Lung Cancer Mortality in the United States From 2015 to 2065: A Comparative Modeling Approach. Annals of internal medicine. 2018;169(10):684–93. Epub 2018/10/12. 10.7326/M18-1250 . 30304504 PMC6242740

[10] Yang G. Tobacco Control in China. Beijing: Beijing Union Medical University Press; 2018.

[11] Qi F, Jia XR, Li SP, Liu H, YN W. Investigation and analysis of tobacco epidemic characteristics and attitudes towards smoking ban among urban and rural residents over 15 years in Qingdao. Chinese Journal of Preventive Medicine. 2016;07:652–5. 10.3760/cma.j.issn.0253-9624.2016.07.017 27412845

[12] Qi F, Xu Z.S., Jia X.R., Lin P., Geng M.Y., Wang Y.N., et al Burden of disease attributable to cigarette smoking in Qingdao in 2015. Chinese Journal of Disease Control & Prevention. 2018;22(04):354–7+85.

[13] Forouzanfar MH, Alexander L, Anderson HR, Bachman VF, Biryukov S, Brauer M, et al Global, regional, and national comparative risk assessment of 79 behavioural, environmental and occupational, and metabolic risks or clusters of risks in 188 countries, 1990–2013: a systematic analysis for the Global Burden of Disease Study 2013. Lancet (London, England). 2015;386(10010):2287–323. 10.1016/S0140-6736(15)00128-2 . 26364544 PMC4685753

[14] Xu Z, Qi F, Wang Y, Jia X, Lin P, Geng M, et al Cancer mortality attributable to cigarette smoking in 2005, 2010 and 2015 in Qingdao, China. PloS one. 2018;13(9). Epub 2018/09/21. 10.1371/journal.pone.0204221 . 30235293 PMC6157816

[15] Peto R, Lopez AD, Boreham J, Thun M, Heath C. Mortality from tobacco in developed countries: indirect estimation from national vital statistics. Lancet (London, England). 1992;339(8804):1268–78. 10.1016/0140-6736(92)91600-d . 1349675

[16] Box GEP, Jenkins G. Time series analysis forecasting and control. 2nd ed San Francisco: Holden-Day; 1976.

[17] Qi F J X.R., Li S.P., Liu H., Wang Y.N. Investigation on the tobacco epidemic and smoking attitudes among residents at early tobacco control legislation in Qingdao city. Chinese Journal of Preventive Medicine. 2016;50(7):652–5. 10.3760/cma.j.issn.0253-9624.2016.07.017 27412845

[18] Yang T, Xu X, Rockett IR, Guo W, Zhou H. Effects of household, workplace, and public place smoking restrictions on smoking cessation. Health Place. 2011;17(4):954–60. Epub 2011/05/10. 10.1016/j.healthplace.2011.04.003 . 21550837

[19] Li Y, Zeng X, Liu J, Liu Y, Liu S, Yin P, et al Can China achieve a one-third reduction in premature mortality from non-communicable diseases by 2030? BMC Med. 2017;15(1):132 Epub 2017/07/12. 10.1186/s12916-017-0894-5 . 28693510 PMC5504650

[20] Kuipers MA, Nagelhout GE, Willemsen MC, Kunst AE. Widening educational inequalities in adolescent smoking following national tobacco control policies in the Netherlands in 2003: a time-series analysis. Addiction. 2014;109(10):1750–9. Epub 2014/06/05. 10.1111/add.12637 . 24895015

[21] Lee YO, Hebert CJ, Nonnemaker JM, Kim AE. Multiple tobacco product use among adults in the United States: Cigarettes, cigars, electronic cigarettes, hookah, smokeless tobacco, and snus. Preventive medicine. 2014;62:14–9. 10.1016/j.ypmed.2014.01.014 24440684

[22] Lee YO, Hebert CJ, Nonnemaker JM, Kim AE. Youth Tobacco Product Use in the United States. Pediatrics. 2015;135(3):409–15. 10.1542/peds.2014-3202 25647680

[23] Chen LS, Hurng BS. The secular trends of disease burden attributed to tobacco smoke in Taiwan 1990–2013. J Formos Med Assoc. 2018;117(1):3–5. Epub 2017/05/28. 10.1016/j.jfma.2017.05.003 . 28549590

[24] Siegel RL, Miller K.D., Jemal A. Cancer statistics, 2018. CA Cancer J Clin 2018;68(1):7–30. 10.3322/caac.21442 29313949

[25] Feliu A, Filippidis FT, Joossens L, Fong GT, Vardavas CI, Baena A, et al Impact of tobacco control policies on smoking prevalence and quit ratios in 27 European Union countries from 2006 to 2014. Tob Control. 2019;28(1):101–9. Epub 2018/02/24. 10.1136/tobaccocontrol-2017-054119 . 29472445 PMC6317447

[26] Liang XF. Report of China City Adult Tobacco Survey 2013–14. Atlanta, Georgia,USA: CDC Foundation; 2015.

[27] Yang J. The Survey Report on Enfcorement of Smoke Free Law in Cities of China. Beijing: China Democracy and Law Press; 2015.

